# Stratification of Wilms tumor by genetic and epigenetic analysis

**DOI:** 10.18632/oncotarget.468

**Published:** 2012-03-31

**Authors:** Richard H. Scott, Anne Murray, Linda Baskcomb, Clare Turnbull, Chey Loveday, Reem Al-Saadi, Richard Williams, Fin Breatnach, Mary Gerrard, Juliet Hale, Janice Kohler, Pablo Lapunzina, Gill A. Levitt, Sue Picton, Barry Pizer, Milind D. Ronghe, Heidi Traunecker, Denise Williams, Anna Kelsey, Gordan M. Vujanic, Neil J. Sebire, Paul Grundy, Charles A. Stiller, Kathy Pritchard-Jones, Jenny Douglas, Nazneen Rahman

**Affiliations:** ^1^ Division of Genetics & Epidemiology, Institute of Cancer Research and Royal Marsden Hospital, Sutton, UK; ^2^ The Wellcome Trust/Cancer Research UK Gurdon Institute, University of Cambridge, Cambridge, UK; ^3^ Division of Molecular Pathology, Institute of Cancer Research and Royal Marsden Hospital, Sutton, UK; ^4^ Department of Paediatric Oncology, Our Lady's Children's Hospital, Dublin, Ireland; ^5^ Department of Paediatric Oncology, Sheffield Children's Hospital, Sheffield, UK; ^6^ Department of Paediatric Oncology, Royal Victoria Infirmary, Newcastle Upon Tyne, UK; ^7^ Department of Paediatric Oncology, Southampton General Hospital, Southampton, UK; ^8^ Department of Genetics, Hospital Universitario La Paz, Madrid, Spain; ^9^ Department of Haematology/Oncology, Great Ormond Street Hospital, London, UK; ^10^ Regional Paediatric Oncology Unit, St James's University Hospital, Leeds, UK; ^11^ Department of Paediatric Oncology, Alder Hey Children's Hospital, Liverpool, UK; ^12^ Schiehallion Unit, Royal Hospital for Sick Children, Glasgow, UK; ^13^ Department of Paediatric Oncology, Children's Hospital for Wales, Cardiff, UK; ^14^ Department of Paediatric Oncology, Addenbrooke's NHS Trust, Cambridge, UK; ^15^ Department of Paediatric Pathology, Royal Manchester Children's Hospital, Manchester, UK; ^16^ Department of Histopathology, School of Medicine, Cardiff University, Cardiff, UK; ^17^ Department of Histopathology, Great Ormond Street Hospital, London, UK; ^18^ Pediatric Hematology, Stollery Children's Hospital, Edmonton, Canada; ^19^ Childhood Cancer Research Group, Department of Paediatrics, University of Oxford, Oxford, UK; ^20^ Molecular Haematology and Cancer Biology Unit, University College London, Institute of Child Health, London, UK

**Keywords:** Wilms tumor, WT1, WTX, CTNNB1, TP53, 11p15, somatic genetic mutation, epigenetic

## Abstract

Somatic defects at five loci, *WT1*, *CTNNB1*, *WTX*, *TP53* and the imprinted 11p15 region, are implicated in Wilms tumor, the commonest childhood kidney cancer. In this study we analysed all five loci in 120 Wilms tumors. We identified epigenetic 11p15 abnormalities in 69% of tumors, 37% were H19 epimutations and 32% were paternal uniparental disomy (pUPD). We identified mutations of *WTX* in 32%, *CTNNB1* in 15%, *WT1* in 12% and *TP53* in 5% of tumors. We identified several significant associations: between 11p15 and *WTX* (P=0.007), between *WT1* and *CTNNB1* (P<0.001), between *WT1* and *pUPD* 11p15 (P=0.01), and a strong negative association between *WT1* and *H19* epimutation (P<0.001). We next used these data to stratify Wilms tumor into three molecular Groups, based on the status at 11p15 and *WT1*. Group 1 tumors (63%) were defined as 11p15-mutant and *WT1*-normal; a third also had *WTX* mutations. Group 2 tumors (13%) were *WT1*-mutant. They either had 11p15 pUPD or were 11p15-normal. Almost all had *CTNNB1* mutations but none had *H19* epimutation. Group 3 tumors (25%) were defined as 11p15-normal and *WT1*-normal and were typically normal at all five loci (P<0.001). We also identified a novel clinical association between *H19* epimutation and bilateral disease (P<0.001). These data provide new insights into the pattern, order, interactions and clinical associations of molecular events in Wilms tumor.

## INTRODUCTION

Wilms tumor is the commonest childhood kidney cancer and affects 1 in 10,000 children [1]. 5% of individuals have bilateral tumors affecting both kidneys [2]. Most Wilms tumors occur in otherwise well children. Approximately 5% of such children have underlying constitutional mutations at *WT1* or epigenetic defects at chromosome 11p15 that predispose to Wilms tumor [3]. Over the past 25 years extensive research has implicated somatic abnormalities at five loci in Wilms tumorigenesis. These are mutations in *WT1*, *CTNNB1*, *WTX*, *TP53* and epigenetic 11p15 abnormalities at the imprinted *H19*/*IGF2* locus [4-11].

Despite the considerable number of Wilms tumors that have been collected through international trials [12-15], limited systematic molecular profiling of these loci has been performed. A major constraint has been the difficulty in undertaking epigenetic analyses at 11p15. Two principal somatic abnormalities occur at 11p15: paternal uniparental disomy (pUPD 11p15) and *H19* epimutation, also known as *IGF2* loss of imprinting [9, 10, 16-19]. These defects result in *H19* hypermethylation and biallelic *IGF2* expression. In addition, somatic copy number defects at 11p15 such as maternal deletions and paternal duplications are reported in a small number of tumors. Comprehensive 11p15 analysis to detect these abnormalities has historically required large amounts of tumor sample and multiple, technically demanding assays. Thus, although 11p15 defects are by far the commonest abnormality in Wilms tumor, disrupted in 50-75% of tumors, 11p15 has often not been analysed to completion [9, 10, 16-19].

To overcome the difficulties of 11p15 profiling, we previously optimised a MS-MLPA (methylation-specific multiplex ligation-dependent probe amplification) assay of 11p15 which allows reliable and comprehensive analysis of both epigenetic and copy number defects in a single analysis that is cost and time-efficient and requires only a small amount of DNA [20, 21]. In this study we performed 11p15 MS-MLPA together with mutation analyses at *WT1*, *CTNNB1*, *WTX* and *TP53* in a series of 120 Wilms tumors to yield new insights into the patterns and interactions of molecular events at the five loci in Wilms tumorigenesis. Our series included 100 ‘sporadic’ tumors in which constitutional defects at *WT1* and 11p15 had been excluded and 20 cases with constitutional *WT1* or 11p15 defects. The latter were included to facilitate investigation into the order of somatic events in tumors as, by definition, the constitutional abnormality is the first event in such tumors.

## RESULTS

### Frequency of somatic mutations in sporadic tumors

In the sporadic tumors, the most common somatic abnormality was at 11p15, which was abnormal in 69% and was due to *H19* epimutation in 37% and pUPD 11p15 in 32%. Monoallelic *WTX* mutations were present in 32% of sporadic tumors. Twenty-one were whole gene deletions and three were truncating point mutations. As expected, the mutations were hemizygous in males and heterozygous in females. Biallelic *WT1* abnormalities were detected in 12% of sporadic tumors and resulted from either a single *WT1* mutation accompanied by UPD for the mutated allele, or two different somatic *WT1* mutations. *CTNNB1* mutations were present in 15% and *TP53* mutations in 5% of sporadic tumors. The frequencies of individual somatic abnormalities are similar to those reported in previous studies [4, 7-11, 16, 17, 19, 22-29]. The full results are given in Supplementary Table 1 and a summary of the results in Table 1.

**Table 1:** Results at the five loci in the 120 Wilms tumors

	Sporadic tumors		All tumors	
	n*	%	n	%
**Any 11p15 abnormality**	**62/90**	**69%**	**81/110**	**74%**
*- H19* epimutation	33	37%	37	34%
- pUPD 11p15	29	32%	44	40%
***WTX*** mutation	**24/74**	**32%**	**29/87**	**33%**
*- WTX* deletion	21	28%	26	30%
- Point mutation	3	4%	3	3%
***WT1*** mutation^†^	**8/66**	**12%**	**17/80**	**21%**
- Mutation and UPD *WT1*	5	8%	12	15%
- Two different mutations	3	5%	5	6%
***CTNNB1*** mutation	**12/79**	**15%**	**19/96**	**20%**
***TP53*** mutation	**3/65**	**5%**	**3/76**	**4%**

* Denominators at each locus are the number of tumors successfully analysed at that locus.

† All *WT1*-mutant tumors had biallelic mutations; this was either one *WT1* mutation followed by UPD or two separate mutations.

### Frequency of mutations in tumors from individuals with constitutional defects

In tumors from individuals with constitutional *WT1* mutation, the wild-type *WT1* allele was somatically inactivated in the tumor, either by UPD or by a somatic mutation. Seven of the nine tumors had *CTNNB1* mutations and the remaining two tumors had *WTX* deletions. In tumors from individuals with constitutional 11p15 defects there was no additional somatic 11p15 defect, as expected. Furthermore, no *WT1* or *CTNNB1* mutations were detected. Three tumors had *WTX* deletions. The full results are given in Supplementary Table 1 and a summary of the results in Table 1.

### Associations between loci

**Figure 1:**
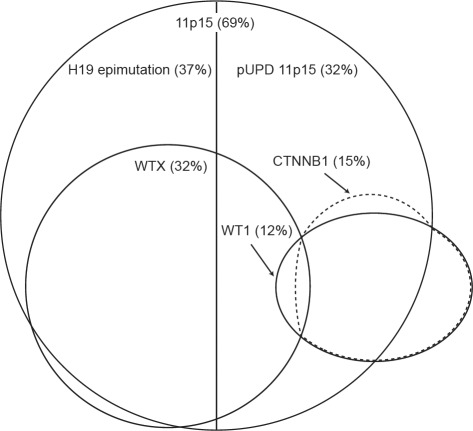
Schematic diagram of the overlapping distributions of molecular abnormalities at 11p15, *WTX*, *WT1* and *CTNNB1* in Wilms tumor The percentage of sporadic tumors with each abnormality in our series is indicated in brackets. *CTNNB1* mutations occur predominantly in tumors with *WT1* mutations. *WTX* mutations occur predominantly in tumors with 11p15 defects. *WT1*-mutant tumors often have pUPD 11p15 while *H19* epimutation, the other class of 11p15 defect, is not seen in this context. *WTX* mutations are infrequent in tumors with *WT1* or *CTNNB1* mutations. (pUPD: paternal uniparental disomy)

We examined the 120 tumors for associations between molecular defects and identified four significant associations between abnormalities at different loci (Figure 1, Supplementary Table 2). Firstly, we identified a strong, novel, positive association between 11p15 and *WTX* defects. *WTX* mutations were significantly more frequent in tumors with 11p15 defects than those without (25/61 vs 2/22, P=0.007), and only two *WTX*-mutant tumors had normal 11p15 status (Table 1). The two subclasses of 11p15 defect, *H19* epimutation and pUPD 11p15, were present in similar proportions of *WTX*-mutant tumors (15/25 vs 10/25). We identified significant associations between 11p15 and *WT1* mutations, but in contrast to *WTX*, the associations differed according to the nature of the 11p15 defect. There was a strong negative association between *WT1* mutation and H19 epimutation, which was not found in any *WT1*-mutant tumor (0/17 vs 24/61 P<0.001) consistent with previous observational data [23]. In contrast there was a positive association between *WT1* mutation and pUPD 11p15 which was present in 12/17 *WT1*-mutant tumors (12/17 vs 21/61 P=0.01). Finally, consistent with multiple previous studies, *CTNNB1* mutations were significantly more frequent in tumors with *WT1* mutations than those without (15/17 vs 2/63, P<0.001) [26, 29, 30]. Each of the three *TP53*-mutant tumors also had *H19* epimutation, but this association did not reach statistical significance.

### Molecular stratification of Wilms tumor into three groups

**Figure 2:**
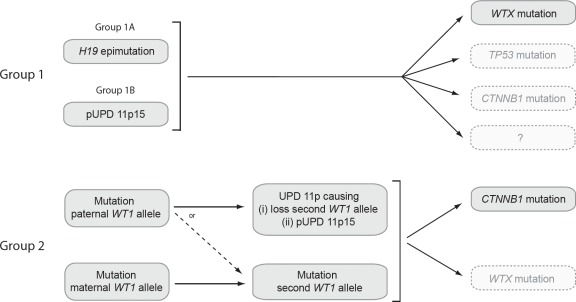
Schematic diagram of the molecular events in tumors in Group 1 and Group 2 Group 1 tumors are defined as having 11p15 defects in the absence of a *WT1* mutation. Group 1 is subdivided into Group 1A, with *H19* epimutation, and Group 1B, with pUPD 11p15. In Group 1 tumors, an 11p15 defect occurs as the likely first event, occurring either constitutionally or somatically. Approximately 30% of Group 1 tumors undergo *WTX* mutation, while mutations in *TP53* and *CTNNB1* mutation are infrequent. No additional event is currently identifiable in the majority of Group 1 tumors. Group 2 tumors are defined as having *WT1* mutations. In Group 2 tumors, monoallelic *WT1* mutation is the likely first event, occurring either constitutionally or somatically. Group 2 tumors in which the mutation targets the paternally-derived *WT1* allele frequently next undergo somatic recombination resulting in UPD 11p and causing loss of the wild-type *WT1* allele and pUPD 11p15. The remainder undergo mutation or deletion of the wild-type *WT1* allele and retain normal 11p15 status. The large majority (~90%) of Group 2 tumors undergo *CTNNB1* mutation, while *WTX* mutation is less common. (pUPD: paternal uniparental disomy; UPD: uniparental disomy)

We examined the patterns of abnormalities at the five loci. This allowed us to partition tumors into molecular groups according to their status at *WT1* and 11p15. The Groups are equally applicable to tumors from sporadic and constitutional cases and both are included in the Group descriptions below. However, it is likely that the relative contributions of the Groups to the sporadic tumors better reflects the pattern in unselected Wilms tumor series (Figure 2, Table 2 and Supplementary Table 1).

**Table 2:** Frequency of abnormalities at the five loci by molecular Group

	Group 1A		Group 1B		Group 2		Group 3	
	n*	%	n	%	n	%	n	%
**Sporadic tumors**	23/64	36%	17/64	27%	8/64	13%	16/64	25%
**All tumors**	24/78	31%	21/78	27%	17/78	22%	16/78	21%
**Any 11p15 abnormality**	24/24	100%	21/21	100%	12/17	71%	0/16	0%
- *H19* epimutation	24/24	100%	0/21	0%	0/17	0%	0/16	0%
- pUPD 11p15	0/24	0%	21/21	100%	12/17	71%	0/16	0%
***WTX*** mutation	10/23	43%	4/21	19%	3/12	25%	0/16	0%
***WT1*** mutation	0/24	0%	0/21	0%	17/17	100%	0/16	0%
***CTNNB1*** mutation	1/24	4%	1/21	5%	15/17	88%	0/16	0%
***TP53*** mutation	3/22	14%	0/21	0%	0/11	0%	0/16	0%

* Denominat ors in each Group are the number of tumors successfully analysed at both 11p15 and *WT1*, the two loci used to classify tumors into Groups. Denominators at each locus are the number of tumors successfully analysed at that locus.

#### Group 1 –11p15-mutant, WT1-normal tumors

We defined Group 1 tumors as having an 11p15 defect but no *WT1* mutation. They were the most common, accounting for 58% of all tumors and 63% of sporadic tumors. We divided Group 1 into two subgroups; Group 1A tumors which have *H19* epimutations (31% of tumors); and Group 1B, which have pUPD 11p15 (27% of tumors). *WTX* mutations were present in 32% of Group 1 tumors. *CTNNB1* mutations were rare, with one *CTNNB1* mutation identified in a Group 1A tumor and one in a Group 1B tumor. *TP53* mutations were found in three Group 1A tumors.

#### Group 2 – WT1-mutant, 11p15 pUPD or 11p15-normal tumors

We defined Group 2 tumors as those with *WT1* mutations. Group 2 accounted for 22% of all tumors and 13% of sporadic tumors. The tumors either had 11p15 pUPD (71%) or were 11p15-normal (29%). *CTNNB1* mutation was present in 88%. *WTX* mutations were seen in a smaller proportion (25%). *H19* epimutation was not found in any Group 2 tumor.

#### Group 3 – 11p15-normal, WT1-normal tumors

We defined Group 3 tumors as having no abnormality at *WT1* or 11p15. They accounted for 25% of sporadic tumors. *CTNNB1, WTX* and *TP53* mutations were not detected in any Group 3 tumor and together were significantly less frequent than in Groups 1 and 2 (0/16 vs 37/62, P<0.001). Thus, the Group 3 tumors were poly-negative for abnormalities at all five loci.

### Clinical associations of molecular groups

The clinical associations of constitutional defects are well established and we therefore focussed our analyses on evaluating the associations in sporadic tumors, in which constitutional *WT1* and 11p15 defects had been excluded. This revealed two interesting, novel, significant associations with younger age at diagnosis and with bilateral disease (Table 3, Supplementary Table 3). Age at diagnosis was significantly lower in sporadic tumors with somatic *WT1* mutations (Group 2), compared with Group 1 and 3 tumors (14 months vs 38 months, P<0.001). Bilateral disease was significantly more frequent in sporadic tumors with somatic *H19* epimutation (Group 1A tumors), than other groups (7/23 vs 0/41, P<0.001). Furthermore, *H19* epimutation was present in three of four additional bilateral cases which could not be classified to a molecular Group because of failed *WT1* analysis. Thus, 10 of 11 cases with bilateral tumors without constitutional mutations had somatic *H19* epimutation (P<0.001). In the two individuals where tumor was available from both kidneys the *H19* epimutation was present bilaterally.

**Table 3:** Frequency of bilateral disease and median age at diagnosis of sporadic tumors in the three molecular Groups

	Total	Bilateral		Age at diagnosis (months)
	n	n	%	median
**All tumors classified**	64	7	11%	38
**Group 1**	40	7	18%	42*
1A - *H19* epimutation	23	7	30%*	42
1B - pUPD 11p15	17	0	0%	43
**Group 2**	8	0	0%	14*
**Group 3**	16	0	0%	39

* Statistically significant associations. P values are given in the text. pUPD: paternal uniparental disomy.

## DISCUSSION

Our results identify novel molecular associations and in particular clarify the interactions and timing of 11p15 defects in Wilms tumor. We report a novel, strong association between defects at 11p15 and *WTX* mutations; 93% of *WTX* mutations occurred in tumors with 11p15 defects. Most importantly, we demonstrate that epigenetic 11p15 defects occur in different contexts and in (at least) two distinct types of Wilms tumor that we call Group 1 and Group 2 tumors. In Group 1 tumors, which are the most common molecular subtype, both classes of 11p15 defect occur. It is probable that in Group 1 tumors the 11p15 defect is an initiating / early event that precedes *WTX* mutation. This is suggested by the occurrence of *WTX* mutation in the tumors with constitutional 11p15 defects, in which it must be a secondary event, and also by the report of individuals with constitutional *WTX* mutations who have a skeletal dysplasia but do not develop Wilms tumor [31].

By contrast, in Group 2 tumors 11p15 defects are secondary rather than initiating events, and only one class of defect, pUPD 11p15, occurs; *H19* epimutation occurs very rarely, if at all. In Group 2 tumors, *WT1* mutation appears to be the initiating / early event (Figure 2). Following *WT1* mutation, a single somatic recombination event causing pUPD encompassing both *WT1* and 11p15 then occurs in a substantial proportion. This will simultaneously inactivate the second *WT1* allele and cause *H19* hypermethylation and biallelic *IGF2* expression. The tumor data from individuals with constitutional defects strongly supports this sequence as eight of nine tumors from individuals with constitutional *WT1* mutations had somatic pUPD 11p15, whereas none of 11 individuals with constitutional 11p15 defects had somatic *WT1* mutations.

A notable observation that emerged from these analyses is that Group 3 tumors, which are defined as those with normal status at both *WT1* and 11p15, were typically also negative for mutations at *WTX*, *CTNNB1* and *TP53* (P<0.001, Table 2). In our series this poly-negative group accounts for ~25% of tumors. The underlying molecular abnormalities in these tumors are currently unknown and research focussed on Group 3 tumors may be particularly fruitful in the discovery of further Wilms tumor genes.

We detected two novel, significant, clinical associations, primarily because we exhaustively discriminated tumors with constitutional 11p15 and *WT1* defects from sporadic tumors. This has rarely been undertaken systematically in previous molecular analyses of Wilms tumors. The first significant association was between sporadic bilateral disease and sporadic Group 1A tumors; 10 of 11 cases with sporadic bilateral tumors had somatic *H19* epimutation (P<0.001). A possible explanation is that the 11p15 defects that drive these tumors occur as early post-zygotic events, and can thus be present in both kidneys but absent from lymphocytes. This may represent a form of tissue-specific cancer predisposition that is intermediate between classic constitutional cancer predisposition and tumor-restricted, somatic events. If so, it may have relevance to other bilateral / multifocal cancers. It is noteworthy that the association with sporadic bilateral tumors was restricted to *H19* epimutation and did not extend to pUPD 11p15. This may indicate that either the risk of Wilms tumor is higher in *H19* epimutation than in pUPD 11p15 tumors and/or that *H19* epimutation is more likely to occur earlier in embryogenesis and hence be present in both kidneys.

The second clinical association that emerged from our data is between early age of diagnosis and sporadic Group 2 tumors, i.e. tumors with somatic but not constitutional *WT1* mutations. It is well established that most constitutional cancer syndrome mutations, including *WT1*, are associated with younger age of onset. However, this association is generally believed to be restricted to tumors with constitutional mutations, rather than their somatically driven counterparts. This may not be the case for Wilms tumors with *WT1* mutations, as our analyses demonstrate that the median age of onset in children with somatic *WT1* mutations was 14 months. This is significantly lower than the other groups, and the median age of sporadic Wilms tumor (38 months) and is similar to tumors in children with constitutional *WT1* mutations [3, 32-34]. The biological explanation for this association is obscure, but it may indicate that *WT1* mutations are associated with a more rapid progression to Wilms tumor than other molecular abnormalities.

In summary, through analysis of a series of Wilms tumors for molecular defects at the five known Wilms tumor genetic loci, this study provides new insights into the sequence and patterns of events that occur during Wilms tumorigenesis. Similar stratification of tumors in large, ongoing clinical trials will allow evaluation of these associations in larger numbers with richer clinic-pathological data, and will likely result in further advances in our understanding of the molecular processes that result in Wilms tumor and their clinical manifestations.

## METHODS

### Samples

We included 120 individuals affected with Wilms tumor recruited by the Wilms Tumor Susceptibility Collaboration (WTSC) as part of the Factors Associated with Childhood Tumors (FACT) study. The collaborators in the WTSC are given in the Supplementary Note. The series consisted of 100 sporadic tumors from children with non-syndromic Wilms tumor from the UK in whom constitutional predisposing defects at *WT1* and 11p15 had been excluded, 9 tumors from individuals with constitutional *WT1* mutations and 11 from individuals with constitutional 11p15 defects (Supplementary Table 1). Patients and/or their families gave consent for the research, which has multicenter research ethics approval (MREC05/02/17).

From each case, genomic DNA was extracted from tumor and peripheral blood lymphocytes using standard techniques. Tumor samples were supplied by the recruiting centre for DNA extraction either as fresh frozen or paraffin embedded tumor. In all cases, a histological section corresponding to the extracted specimen was reviewed by a single pathologist. Cases were included in the study only if this review confirmed the section to be Wilms tumor and that >80% examined nuclei were tumor. The large majority of individuals in the series, including all of those with sporadic, non-syndromic disease were treated according to European protocols, in UK Paediatric Oncology centres. In these cases, tumor resection therefore followed neoadjuvant chemotherapy (Supplementary Table 3). Staging and histological group of tumors was classified according to the relevant trial protocol [14, 35-37].

### Mutation analysis of *WT1*, *TP53*, *CTNNB1* and *WTX*

We analysed *WT1*, *TP53* and *WTX* by direct sequencing of their full coding sequences and intron-exon boundaries in 10, seven and 11 fragments respectively. We analysed the mutation hot-spot of *CTNNB1* by sequencing of exon 3 as a single fragment. PCR amplification was performed on native or whole-genome amplified DNA using the primers and conditions specified in Supplementary Table 4. Amplicons were then sequenced using the BigDyeTerminator Cycle Sequencing Kit and an ABI 3730 automated sequencer (Applied Biosystems). Mutations in whole-genome amplified samples were confirmed in native DNA. Sequence traces were analysed using Mutation Surveyor software v3.20 (SoftGenetics) and by visual inspection. Only samples in which >90% of the coding sequence of a gene was successfully screened were considered to have passed at that locus. We also performed dosage analysis at *WT1* and *WTX* by MLPA (multiplex ligation-dependent probe amplification) according to the manufacturer's instructions using a customised version of the P118 *WT1* kit (MRC Holland) and an ABI 3130 automated sequencer. The P118 kit contains 11 probes targeting the 10 exons of *WT1* and 13 probes to flanking genes at 11p13. To allow dosage analysis at *WTX*, we customised the kit by adding four synthetic probes targeting the *WTX* coding sequence, two probes targeting the X chromosome outside the *WTX* deletion region and one Y chromosome probe (Supplementary Table 5). MLPA traces were analysed using GeneMarker v1.51 software (SoftGenetics). For *WT1* and *WTX*, samples were required to pass MLPA analysis to pass analysis overall at the locus.

### Epigenetic analysis of 11p15

We analysed the imprinted 11p15 region for the range of reported methylation and copy number defects using methylation-specific MLPA (MS-MLPA) as previously described using a customised version of the ME030 kit (MRC Holland) [20, 21]. The assay determines the level of methylation at two differentially methylated regions (DMRs) at 11p15, *H19* and KvDMR and also copy number at 28 loci across the 11p15 region. Samples were classified as follows: (1) Normal methylation and normal copy number - No 11p15 defect, (2) Abnormal methylation and normal copy number - 11p15 epigenetic defect, subclassified according to pattern of abnormal methylation such that samples with *H19* hypermethylation and KvDMR normal methylation were classified as *H19* epimutation whilst those with *H19* hypermethylation and KvDMR hypomethylation were classified as pUPD 11p15. (3) Abnormal methylation and abnormal copy number - 11p15 copy number defect, deletion (decreased copy number) or duplication (increased copy number); the parent of origin of the abnormal allele is given by the pattern of methylation.

Normal ranges for methylation indices in constitutional samples are as previously published [20, 21]. To account for the clonal nature of tumor DNA, we used more stringent cut-offs for abnormal methylation, requiring abnormal tumor samples to show >70% methylated alleles. In samples with pUPD 11p15, we confirmed the abnormality by analysis of 11p15 microsatellites as previously described [20, 21]. Lymphocyte DNA from the proband and available parents was analysed in parallel with tumor DNA, allowing the parental origin of alleles to be assigned.

Where an abnormality was identified in tumor DNA, we analysed lymphocyte DNA for the same abnormality to differentiate constitutional from somatic events. For abnormalities identified in a tumor from individuals with bilateral disease, we analysed DNA from the contralateral tumor if material was available.

### Statistical analysis

We performed comparisons of the frequencies of abnormalities at different loci and between molecular subgroups using a two-sided Fisher's exact test. We compared the frequencies of bilateral disease and unfavorable histology using a two-sided Fisher's exact test and compared stage and age at diagnosis using the Wilcoxon rank-sum test.

## SUPPLEMENTARY TABLES AND NOTE





## References

[1] Stiller CA, Parkin DM (1990). International variations in the incidence of childhood renal tumours. Br J Cancer.

[2] Breslow NE, Olson J, Moksness J, Beckwith JB, Grundy P (1996). Familial Wilms' tumor: a descriptive study. Med Pediatr Oncol.

[3] Scott RH, Stiller CA, Walker L, Rahman N (2006). Syndromes and constitutional chromosomal abnormalities associated with Wilms tumour. J Med Genet.

[4] Bardeesy N, Falkoff D, Petruzzi MJ, Nowak N, Zabel B, Adam M, Aguiar MC, Grundy P, Shows T, Pelletier J (1994). Anaplastic Wilms' tumour, a subtype displaying poor prognosis, harbours p53 gene mutations. Nat Genet.

[5] Call KM, Glaser T, Ito CY, Buckler AJ, Pelletier J, Haber DA, Rose EA, Kral A, Yeger H, Lewis WH (1990). Isolation and characterization of a zinc finger polypeptide gene at the human chromosome 11 Wilms' tumor locus. Cell.

[6] Gessler M, Poustka A, Cavenee W, Neve RL, Orkin SH, Bruns GA (1990). Homozygous deletion in Wilms tumours of a zinc-finger gene identified by chromosome jumping. Nature.

[7] Koesters R, Ridder R, Kopp-Schneider A, Betts D, Adams V, Niggli F, Briner J, von Knebel Doeberitz M (1999). Mutational activation of the beta-catenin proto-oncogene is a common event in the development of Wilms' tumors. Cancer Res.

[8] Malkin D, Sexsmith E, Yeger H, Williams BR, Coppes MJ (1994). Mutations of the p53 tumor suppressor gene occur infrequently in Wilms' tumor. Cancer Res.

[9] Ogawa O, Eccles MR, Szeto J, McNoe LA, Yun K, Maw MA, Smith PJ, Reeve AE (1993). Relaxation of insulin-like growth factor II gene imprinting implicated in Wilms' tumour. Nature.

[10] Rainier S, Johnson LA, Dobry CJ, Ping AJ, Grundy PE, Feinberg AP (1993). Relaxation of imprinted genes in human cancer. Nature.

[11] Rivera MN, Kim WJ, Wells J, Driscoll DR, Brannigan BW, Han M, Kim JC, Feinberg AP, Gerald WL, Vargas SO, Chin L, Iafrate AJ, Bell DW, Haber DA (2007). An X chromosome gene, WTX, is commonly inactivated in Wilms tumor. Science.

[12] Green DM, Breslow NE, Beckwith JB, Finklestein JZ, Grundy P, Thomas PR, Kim T, Shochat S, Haase G, Ritchey M, Kelalis P, D'Angio GJ (1998). Effect of duration of treatment on treatment outcome and cost of treatment for Wilms' tumor: a report from the National Wilms' Tumor Study Group. J Clin Oncol.

[13] Grundy PE, Breslow NE, Li S, Perlman E, Beckwith JB, Ritchey ML, Shamberger RC, Haase GM, D'Angio GJ, Donaldson M, Coppes MJ, Malogolowkin M, Shearer P, Thomas PR, Macklis R, Tomlinson G (2005). Loss of heterozygosity for chromosomes 1p and 16q is an adverse prognostic factor in favorable-histology Wilms tumor: a report from the National Wilms Tumor Study Group. J Clin Oncol.

[14] Mitchell C, Pritchard-Jones K, Shannon R, Hutton C, Stevens S, Machin D, Imeson J, Kelsey A, Vujanic GM, Gornall P, Walker J, Taylor R, Sartori P, Hale J, Levitt G, Messahel B (2006). Immediate nephrectomy versus preoperative chemotherapy in the management of non-metastatic Wilms' tumour: results of a randomised trial (UKW3) by the UK Children's Cancer Study Group. Eur J Cancer.

[15] Pritchard-Jones K, Pritchard J (2004). Success of clinical trials in childhood Wilms' tumour around the world. Lancet.

[16] Moulton T, Chung WY, Yuan L, Hensle T, Waber P, Nisen P, Tycko B (1996). Genomic imprinting and Wilms' tumor. Med Pediatr Oncol.

[17] Moulton T, Crenshaw T, Hao Y, Moosikasuwan J, Lin N, Dembitzer F, Hensle T, Weiss L, McMorrow L, Loew T (1994). Epigenetic lesions at the H19 locus in Wilms' tumour patients. Nat Genet.

[18] Okamoto K, Morison IM, Taniguchi T, Reeve AE (1997). Epigenetic changes at the insulin-like growth factor II/H19 locus in developing kidney is an early event in Wilms tumorigenesis. Proc Natl Acad Sci U S A.

[19] Steenman MJ, Rainier S, Dobry CJ, Grundy P, Horon IL, Feinberg AP (1994). Loss of imprinting of IGF2 is linked to reduced expression and abnormal methylation of H19 in Wilms' tumour. Nat Genet.

[20] Scott RH, Douglas J, Baskcomb L, Huxter N, Barker K, Hanks S, Craft A, Gerrard M, Kohler JA, Levitt GA, Picton S, Pizer B, Ronghe MD, Williams D, Cook JA, Pujol P (2008). Constitutional 11p15 abnormalities, including heritable imprinting center mutations, cause nonsyndromic Wilms tumor. Nat Genet.

[21] Scott RH, Douglas J, Baskcomb L, Nygren AO, Birch JM, Cole TR, Cormier-Daire V, Eastwood DM, Garcia-Minaur S, Lupunzina P, Tatton-Brown K, Bliek J, Maher ER, Rahman N (2008). Methylation-specific multiplex ligation-dependent probe amplification (MS-MLPA) robustly detects and distinguishes 11p15 abnormalities associated with overgrowth and growth retardation. J Med Genet.

[22] Forbes S, Clements J, Dawson E, Bamford S, Webb T, Dogan A, Flanagan A, Teague J, Wooster R, Futreal PA, Stratton MR (2006). COSMIC 2005. Br J Cancer.

[23] Fukuzawa R, Anaka MR, Weeks RJ, Morison IM, Reeve AE (2009). Canonical WNT signalling determines lineage specificity in Wilms tumour. Oncogene.

[24] Fukuzawa R, Breslow NE, Morison IM, Dwyer P, Kusafuka T, Kobayashi Y, Becroft DM, Beckwith JB, Perlman EJ, Reeve AE (2004). Epigenetic differences between Wilms' tumours in white and east-Asian children. Lancet.

[25] Gessler M, Konig A, Arden K, Grundy P, Orkin S, Sallan S, Peters C, Ruyle S, Mandell J, Li F (1994). Infrequent mutation of the WT1 gene in 77 Wilms' Tumors. Hum Mutat.

[26] Maiti S, Alam R, Amos CI, Huff V (2000). Frequent association of beta-catenin and WT1 mutations in Wilms tumors. Cancer Res.

[27] Ruteshouser EC, Robinson SM, Huff V (2008). Wilms tumor genetics: mutations in WT1, WTX, and CTNNB1 account for only about one-third of tumors. Genes Chromosomes Cancer.

[28] Satoh Y, Nakadate H, Nakagawachi T, Higashimoto K, Joh K, Masaki Z, Uozumi J, Kaneko Y, Mukai T, Soejima H (2006). Genetic and epigenetic alterations on the short arm of chromosome 11 are involved in a majority of sporadic Wilms' tumours. Br J Cancer.

[29] Wegert J, Wittmann S, Leuschner I, Geissinger E, Graf N, Gessler M (2009). WTX inactivation is a frequent, but late event in Wilms tumors without apparent clinical impact. Genes Chromosomes Cancer.

[30] Fukuzawa R, Heathcott RW, Sano M, Morison IM, Yun K, Reeve AE (2004). Myogenesis in Wilms' tumors is associated with mutations of the WT1 gene and activation of Bcl-2 and the Wnt signaling pathway. Pediatr Dev Pathol.

[31] Jenkins ZA, van Kogelenberg M, Morgan T, Jeffs A, Fukuzawa R, Pearl E, Thaller C, Hing AV, Porteous ME, Garcia-Minaur S, Bohring A, Lacombe D, Stewart F, Fiskerstrand T, Bindoff L, Berland S (2009). Germline mutations in WTX cause a sclerosing skeletal dysplasia but do not predispose to tumorigenesis. Nat Genet.

[32] Breslow N, Olshan A, Beckwith JB, Green DM (1993). Epidemiology of Wilms tumor. Med Pediatr Oncol.

[33] Breslow NE, Norris R, Norkool PA, Kang T, Beckwith JB, Perlman EJ, Ritchey ML, Green DM, Nichols KE (2003). Characteristics and outcomes of children with the Wilms tumor-Aniridia syndrome: a report from the National Wilms Tumor Study Group. J Clin Oncol.

[34] Royer-Pokora B, Beier M, Henzler M, Alam R, Schumacher V, Weirich A, Huff V (2004). Twenty-four new cases of WT1 germline mutations and review of the literature: genotype/phenotype correlations for Wilms tumor development. Am J Med Genet A.

[35] Mitchell C, Jones PM, Kelsey A, Vujanic GM, Marsden B, Shannon R, Gornall P, Owens C, Taylor R, Imeson J, Middleton H, Pritchard J (2000). The treatment of Wilms' tumour: results of the United Kingdom Children's cancer study group (UKCCSG) second Wilms' tumour study. Br J Cancer.

[36] Pritchard J, Imeson J, Barnes J, Cotterill S, Gough D, Marsden HB, Morris-Jones P, Pearson D (1995). Results of the United Kingdom Children's Cancer Study Group first Wilms' Tumor Study. J Clin Oncol.

[37] Vujanic GM, Sandstedt B, Harms D, Kelsey A, Leuschner I, de Kraker J (2002). Revised International Society of Paediatric Oncology (SIOP) working classification of renal tumors of childhood. Med Pediatr Oncol.

