# Supplementary material for: Stratification of Wilms tumor by genetic and epigenetic analysis
Source: Oncotarget. 2012 Mar 31;3(3):327–35. doi: 10.18632/oncotarget.468 (PMC3359888; doi:10.18632/oncotarget.468)
Supplement: Supplementary file 2 [file oncotarget-03-327-s002.pdf]

Stratification of Wilms tumor by genetic and epigenetic analysis:

Supplementary Note

The Wilms Tumor Susceptibility Collaboration (WTSC)

The WTSC undertakes recruitment of families and collection of samples from UK patients with Wilms tumor. It is a subgroup of the Factors Associated with Childhood Tumors (FACT) Study. It includes the following collaborators:

J. Anderson, R. Armstrong, A. Atra, J. Barwell, J. Birch, P. Brock, A. Burke, J. Chisholm, D. Cilliers, M. Cohen, T. Cole, N. Coleman, J. Cook, R. Cox, M. Critchfield, D. Eccles, M. Elliott, S. Elkins, P. Fagelman, R. Fisher, N. Foulds, D. Galvin, M. Gerrard, A. Glaser, J. Gray, J. Hale, R. Grundy, D. Halliday, D. Hargrave, D. Heney, T. Homfray, L. Hook, K. Howe, L. Howell, N. Hubbard, A. Jenkins, M. Jenney, G. Jenner, F. Jessop, J. Kingston, S. Knight, J. Kohler, D. Lancaster, G. Levitt, I. Lewis, S. Lowis, A. Lucassen, P. Lunt, E. Mackie, A. Magee, E. Maher, E. McCann, C. McConville, H. McDowell, M. McEntagart, S. Mellor, C. Mercer, A. Michalski, C. Mitchell, B. Morland, C. Morley-Jacob, D. Murphy, A. Murray, A. Ng, J. Nicholson, G. Nicolin, C. Novorol, J. Payne, A. Pearson, R. Phillips, B. Phillips, S. Picton, B. Pizer, J. Powell, K. Pritchard-Jones, M. Radford, R. Ramanujachar, H. Rees, U. Reid, M. Ronghe, E. Roper, E. Ross, R. Sandford, P. Sartori, R. Scott, S. Shanley, R. Shannon, E. Sheridan, E. Simpson, O. Slater, S. Smithson, R. Spicer, M. Stevens, M. Suri, S. Thomas, S. Tomkins, H. Traunecker, J. Tunnacliffe, S. Vaidya, P. Vasudevan, J. Visser, G. Vujanic, D. Walker, C. Waner, K. Wheeler, D. Williams, D. Yeomanson
